# Flexible Resistive Sensors for Wearable and Ergonomics Applications: A Systematic Review

**DOI:** 10.3390/s26082563

**Published:** 2026-04-21

**Authors:** Mina Tabrizi, Ignacio Gil, Montserrat Corbalan, Raúl Fernández-García

**Affiliations:** Department of Electronic Engineering, Universitat Politecnica de Catalunya, 08222 Barcelona, Spain; mina.tabrizi@upc.edu (M.T.); ignasi.gil@upc.edu (I.G.); montserrat.corbalan@upc.edu (M.C.)

**Keywords:** textile, fabric, wearable, resistive, flexible, sensor, sensitivity, ergonomics

## Abstract

Flexible resistive sensors are promising for wearable and ergonomic applications because they can be easily fabricated on textiles or flexible substrates and enable real-time monitoring of human movement and posture, especially in health monitoring systems. This review presents an overview of recent developments in an interdisciplinary way and summarises advances in materials, fabrication methods, and ergonomic applications. A structured literature search was conducted across major databases, including only studies focused on resistive sensing. The selected works were analysed in terms of conductive materials, fabrication techniques (e.g., direct ink writing (DIW) and textile-based methods), and their integration into wearable systems. Flexible resistive sensors are widely used for monitoring joint motion, posture, and physiological signals in healthcare and industrial environments. However, several challenges remain, including limitations in sensitivity, signal stability, material durability, and the need for reliable calibration in real-world conditions. This review highlights current progress and existing limitations and outlines future research directions toward more robust and user-friendly wearable sensing solutions for ergonomic applications.

## 1. Introduction

Musculoskeletal disorders (MSDs) represent a major global health concern, affecting quality of life, productivity, and healthcare systems. Epidemiological studies show that MSDs are highly prevalent across different working populations, particularly those exposed to prolonged sitting and poor postural habits. For example, among sedentary professionals, MSDs are most frequently reported in the lower back (52%), neck (45%), and upper back (38%) [1]. Similarly, professional drivers are exposed to additional risk factors such as whole-body vibration, constrained postures, and long working hours, leading to MSD prevalence rates as high as 86.8% [2]. These findings highlight the urgent need for objective, continuous, and real-time monitoring of human posture in both occupational and daily life environments.

To design and develop flexible resistive sensors for ergonomic applications, it is essential to understand the health problems they aim to address. Beyond posture assessment methods such as REBA (Rapid Entire Body Assessment) [3] and RULA (Rapid Upper Limb Assessment) [4], gait and movement analysis are also important tools for understanding human health and functional performance. Gait parametres, such as pressure distribution, provide valuable insights into musculoskeletal function, rehabilitation progress, and overall mobility, making gait analysis a promising predictive indicator of future health outcomes [5,6]. Therefore, integrating posture and gait monitoring into wearable systems can support the early detection of fatigue, injury risk, and biomechanical abnormalities, enabling more effective preventive and personalised healthcare strategies [5,6,7].

In the field of wearable electronics, flexible resistive sensors are often used to measure changes in electrical resistance caused by different physical inputs, such as strain [8], pressure [9], temperature [10,11], or humidity [12]. Constructed from materials that can stretch, bend, and conform to various shapes, they have become essential in health and wearable applications due to their low-profile integration and cost. These sensors play a key role in devices designed for ergonomic monitoring. Their flexibility allows seamless integration into fabrics, offering real-time measurement of movement and posture in joints, like finger joints, wrists, and elbows. Resistive flexible sensors are both cost-effective and easily embedded in fabrics or flexible substrates [8,9,13,14,15].

These sensors are especially crucial in applications where continuous, real-time monitoring is necessary, such as in space missions [16], healthcare [10,17], and driver fatigue detection systems [18]. For example, astronauts require real-time biochemical monitoring to track their physical condition during missions, ensuring that any strain or abnormal posture can be quickly corrected to prevent long-term injury or fatigue. Wearables equipped with flexible resistive sensors can help prevent injuries and improve training by providing feedback on muscle exertion or poor posture. The use of resistive flexible sensors in ergonomics provides a significant advantage over traditional sensors due to their adaptability and precision.

This paper reviews the development of flexible resistive sensors for wearable devices aimed at real-time health and posture monitoring. It also examines materials and fabrication techniques suitable for integrating these sensors into fabrics and flexible substrates.

The remainder of this paper is organised as follows. Section 2 introduces fundamental concepts of ergonomics relevant to wearable sensing. Section 3 describes the operating principles of flexible resistive sensors. Section 4 outlines the search strategy and selection criteria used in this review. Section 5 presents commonly used substrate and conductive materials, while Section 6 discusses fabrication techniques. Section 7 reviews ergonomic applications of flexible resistive sensors. Finally, Section 8 summarises the main conclusions and future research directions.

## 2. Ergonomics Fundamentals

Ergonomics, also known as Human Factors Engineering [19], is the scientific discipline concerned with the understanding of the interactions among humans and other elements of a system [19]. In automotive design, ergonomics focuses on optimising driver posture, control reachability, and field of view, while minimising physical strain and enhancing safety during vehicle operation [20].

Effective ergonomic analysis requires measurements that reflect the body’s behaviour during movement, rather than relying only on static dimensions. Anthropometric parametres such as limb length or joint angles and arm or chest circumference (meaning how wide a limb or chest becomes during movement) can change significantly during dynamic activities (e.g., lifting the arms or bending forward). These dynamic variations, which are different from static postures, influence the fit, comfort, and performance of ergonomic clothing and wearable devices. Consequently, consideration of dimensional changes across different body regions and motion types is essential for improving accuracy and usability, whereas reliance on static data alone may lead to poor fit and limited functionality in real-world applications [21].

Traditional ergonomic assessment methods, including REBA (Rapid Entire Body Assessment) [3], RULA (Rapid Upper Limb Assessment) [4], and OWAS (Ovako Working Posture Analysis System) [22], are primarily based on manual observation and expert judgement. More recently, image-based motion capture techniques such as OpenPose [3] have been used to identify body segments and automatically select appropriate assessment methods based on measured joint angles [22]. These approaches improve objectivity and enable continuous posture monitoring. In addition, machine learning techniques can predict ergonomic risk levels directly from three-dimensional joint coordinates, allowing automated classification of posture-related musculoskeletal risks [23]. However, such methods largely rely on posture recognition and cannot directly quantify mechanical deformation, strain distribution, or load interaction between the human body and external surfaces, highlighting the need for wearable sensing technologies for comprehensive ergonomic monitoring.

**Figure 1:**
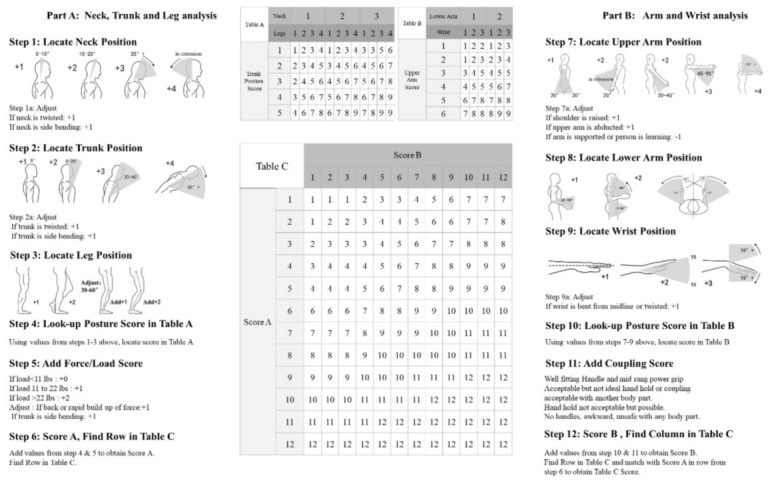
Body segments evaluated in the REBA method for whole-body ergonomic risk assessment [3].

Some ergonomic assessment methods are based on biomechanical parametres that describe human posture and movement during physical activities. As shown in Figure 1, the REBA method evaluates whole-body postural risk by scoring the positions of major body segments, including the neck, trunk, legs, upper arms, lower arms, and wrists [3]. Scores are assigned according to the deviation of each segment from a neutral posture (i.e., by comparing observed joint angles), with additional adjustments for factors such as load, coupling quality, activity level, and activity frequency. These values are combined using standardised lookup tables to obtain a final score representing the overall level of musculoskeletal risk. According to REBA guidelines, a score of 1 indicates negligible risk, 2–3 indicates low risk, 4–7 indicates moderate risk, 8–10 indicates high risk, and 11–15 indicates very high risk requiring prompt corrective action [3].

In real-world settings, particularly in sedentary occupations, significant ergonomic risks persist despite the availability of assessment methods. Understanding ergonomics and biomechanics is therefore essential for designing flexible resistive sensors for joint and posture monitoring [24,25], especially for populations exposed to prolonged sitting and poor posture, such as professional drivers [26]. Ergonomic factors, including usability, comfort, and user trust, strongly influence long-term adoption of wearable devices [19,21], while individual characteristics (e.g., age, activity level, and self-efficacy) also affect user perception and continued use [27]. Sedentary work primarily places strain on the neck, shoulders, back, wrists, and knees, increasing the risk of musculoskeletal disorders due to repetitive movements and sustained postures [26,28].

In the development of integrated wearable devices (IWDs) for health monitoring and wearable applications [29], flexible resistive sensors can measure joint movement and pressure distribution in real time, providing continuous feedback on posture and motion to improve comfort, safety, and user well-being. Such monitoring also enables early detection of fatigue by identifying poor posture or repetitive strain patterns over time. By continuously capturing joint angles, body segment positions, and movement patterns, these sensors support objective quantification of biomechanical exposure. Consequently, ergonomic risk levels can be evaluated using measurable data rather than relying solely on traditional assessment methods applied periodically [19,20,21,30].

## 3. Flexible Resistive Sensor Fundamentals

Resistive flexible sensors operate on the principle of changes in electrical resistance and can detect not only mechanical deformation, such as stretching, bending, or compression, but also environmental factors, like temperature and humidity. While their flexibility is beneficial, the integration of resistive sensors into textiles, flexible substrates, and wearable systems also depends on factors such as sensor structure, material compatibility, and fabrication methods. These sensors offer a seamless way to monitor body movements, posture, and ergonomic conditions in the healthcare, aerospace, and automotive industries.

The main operating principle of flexible resistive sensors is based on the relationship between mechanical deformation and electrical resistance [31]. When a sensor undergoes stretching, compression, or bending, both its length and cross-sectional area may change, altering the resistance of the conductive material. This variation in resistance can be measured and used to quantify the degree of strain or movement. The general formula used to describe this relationship is shown in Equation (1) as follows:

(1) $$R=\rho \frac{L}{A}$$

where *R* is Resistance(in ohms, Ω), rho (ρ) is the electrical resistivity of the material (in ohm-metres, Ω·m), *L* is the length (in metres, m), and *A* is the cross-sectional area (in square metres, m^2^). When a sensor undergoes deformation, such as stretching or compression, both its length (*L*) and cross-sectional area (*A*) change. These changes result in a measurable variation in resistance.

In flexible resistive sensors, this effect is harnessed to detect mechanical deformations. As the sensor is deformed, the geometry of the conductive path changes, resulting in a measurable resistance variation. This change is directly related to the applied strain, allowing the sensor to translate mechanical movements into electrical signals.

The sensitivity of such sensors is often quantified by the gauge factor (GF), defined as Equation (2):

(2) $$GF=\frac{\left(\Delta R/R\right)}{\varepsilon }$$

In this equation,

- GF is the gauge factor, which indicates how sensitive the sensor is to strain (dimensionless).
- ΔR is the change in electrical resistance due to deformation (in ohms, Ω).
- *R* is the original (baseline) resistance (in ohms, Ω).
- ε (strain) is the applied mechanical strain, defined as the relative change in length: $\varepsilon =\frac{\Delta L}{L}$ (dimensionless, often expressed in %).

Understanding these relationships is crucial for designing sensors with desired performance characteristics, especially in applications requiring precise detection of mechanical deformations.

## 4. Search Strategy and Selection Criteria for Scholarly Articles

The process of selecting scholarly articles and conference proceedings involved a thorough search across key databases, such as PubMed, MDPI, ScienceDirect, Scopus, and IEEE Xplore. The search strategy was based on a combination of relevant keywords and their synonyms, related to flexible resistive sensors and ergonomic applications. Keywords such as “textile”, “fabric”, “wearable”, “resistive”, “flexible”, “sensor”, “sensitivity”, and “ergonomics” were used in different combinations to retrieve relevant studies. Boolean operators (AND, OR) were applied where appropriate to refine the search results. The search covered publications from the past two decades. A systematic screening process was then conducted, including the removal of duplicate records and the evaluation of relevance based on predefined inclusion and exclusion criteria.

The criteria for including studies were comprehensive and focused on the following: (1) research on textiles, fabrics, or flexible substrates incorporating resistive elements, particularly in the context of sensor technology, and studies examining their sensitivity; (2) applications of resistive flexible sensors; (3) papers published in English; and (4) studies published in peer-reviewed journals or conferences.

**Figure 2:**
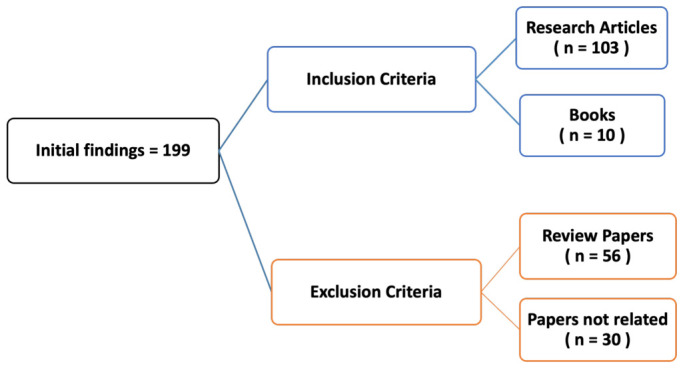
Flowchart of the literature selection process, summarising the identification, exclusion, and categorization of 113 references, including books and research articles related to resistive sensor-based ergonomic and wearable studies.

Exclusion criteria were established to filter out the following: (1) review articles and (2) studies not related to the subject of this review. Following the outlined strategy (Figure 2), the literature was systematically screened, resulting in 199 initial records. After applying the inclusion and exclusion criteria, a total of 113 relevant references were selected for detailed analysis. In addition, this systematic review is based on the PRISMA 2020 guidelines, and the corresponding checklist is provided as Supplementary Material.

## 5. Materials

The choice of materials plays an important role in determining the performance of flexible resistive sensors and their suitability for wearable applications.

Two main components are the substrate, which provides the base structure, and the conductive material, which enables the generation and transmission of electrical signals. Both substrates and conductive materials need to be flexible for the chosen manufacturing process. In addition, conductive materials should provide good electrical performance without losing their ability to bend and stretch, ensuring high gauge factors, stretchability, and durability, qualities that are essential for detecting complex human movements [32,33,34]. The following subsections present an overview of commonly used substrates and conductive materials, outlining their properties, benefits, and limitations in the context of ergonomic and health-related wearable systems.

### 5.1. Substrates

Flexible substrates used in resistive sensors include both polymer and textile materials. Common polymer-based options are polyurethane (PU) [35,36], thermoplastic polyurethane (TPU) [37,38,39], polydimethylsiloxane (PDMS) [40,41], and polyvinyl alcohol (PVA) [42], which are chosen for their flexibility and ease of processing. Textile-based substrates are also widely used because they are breathable, comfortable, and suitable for wearable applications. Examples include commercial fabrics [34,43], cotton-spandex blends [43] , and recycled jeans fabric [10], and silk-based textiles [44], which offer low-cost, sustainable, and biocompatible alternatives for integrating sensors into clothing.

The substrates listed in Table 1 offer different mechanical and functional properties that influence sensor performance. Elastomeric materials like PDMS and TPU provide high stretchability and skin compatibility, making them suitable for wearable and skin-mounted sensors [37,45]. In contrast, PET offers mechanical stability but limited flexibility, often used as a supporting layer in hybrid systems [46]. Textile substrates, such as polyester and cotton–spandex blends, enable direct integration into garments, supporting embroidered or stitched sensor designs for joint and posture monitoring [34,43].

### 5.2. Conductive Materials

Conductive materials vary; the inks used in flexible resistive sensors include metal-based materials such as silver nanoparticles (AgNPs) [49,50,51], silver nanowires (AgNWs) [52,53], silver (Ag) flake [54,55]; conductive polymers such as PEDOT: PSS [56], and polypyrrole (PPy); and carbon-based inks such as carbon nanotubes (CNTs) [35,38,42,57], carbon black (CB) [39], multiwall carbon nanotubes (MWCNTs) [12,37,39,40,58], graphene [59] and reduced graphene oxide (rGO) [44]. Additionally, polyaniline (PANI) [60], conductive threads (stainless steel threads), polybutylene terephthalate (PBT) threads [21], and coated fibre or yarn threads such as nano-silver coated fibres [15,21], copper wire [10], as well as gallium-based alloys [61] are also utilised.

Table 2 presents commonly used conductive materials in flexible resistive sensors, along with their conductivity, stretchability, sensitivity, and typical applications.

These materials offer different balances between electrical performance and mechanical flexibility. Carbon-based materials such as carbon nanotubes (CNTs) [57] and graphene nanoplatelets (GNPs) [46], graphene woven fabrics (GWFs) [30] provide high conductivity and sensitivity, making them ideal for wearable strain [41] and pressure sensors [35,46]. Conductive polymers like PEDOT:PSS and PANI offer good flexibility and biocompatibility, useful for printed and skin-contact applications [56,60]. Metal-based materials, including silver nanoparticles and copper wires, deliver excellent conductivity but are typically less stretchable, making them suitable for rigid or embroidered sensor structures [10,15].

Piezoresistive materials operate by converting mechanical deformation into measurable changes in electrical resistance. In most flexible sensors, the structure consists of a conductive phase dispersed within or deposited onto an elastic substrate (e.g., polymers or elastomers). When mechanical strain or pressure is applied, the conductive network is deformed, resulting in variations in contact resistance, tunnelling distance between conductive fillers, and the configuration of the conductive pathways. These resistance changes correlate with the applied stimulus and form the basis for strain or pressure sensing [13,63,64,65].

## 6. Fabrication Methods

Among the various fabrication approaches, textile-based techniques such as embroidery [34], knitting [66,67], and stitching [68] are widely used for integrating conductive materials into flexible substrates. These methods are particularly suitable for wearable applications due to their comfort, breathability, and compatibility with garment manufacturing. In parallel, printing-based techniques have gained significant attention due to their cost-effectiveness, flexibility, and environmentally friendly characteristics in the production of resistive flexible sensors [33].

Printed sensors offer advantages in mass production, low cost, and customizable patterns on soft substrates [32,69]. By using stabilised nanomaterials with additives and surfactants, printing allows for precise control over material properties and deposition parametres to achieve high-resolution conductive structures and uniform sensing layers [33]. Techniques such as screen and inkjet printing enable the fabrication of versatile sensors on a wide range of substrates. Continued advancements in this field promise innovative solutions for efficient electronic device manufacturing.

The following section reviews textile fabrication methods, as well as screen printing, inkjet printing, and direct ink writing (DIW) techniques for the fabrication of resistive sensors.

### 6.1. Textile Fabrication Methods

Textile-based resistive sensors can be fabricated using various techniques, such as embroidery [10,34], knitting [66,67,70], braiding [70], hand-stitching [43], stitching [62,68]. These methods allow conductive yarns or threads to be integrated directly into fabrics, resulting in flexible and wearable sensor systems. For example, embroidery and stitching have been used to develop sensors for monitoring motion [43,67,71], respiration [43,68], heartbeat [43], and temperature [10]. In one study [34], a silver-coated polyamide thread was integrated into the elastic substrate using embroidery to detect knee-joint movement.

**Figure 3:**
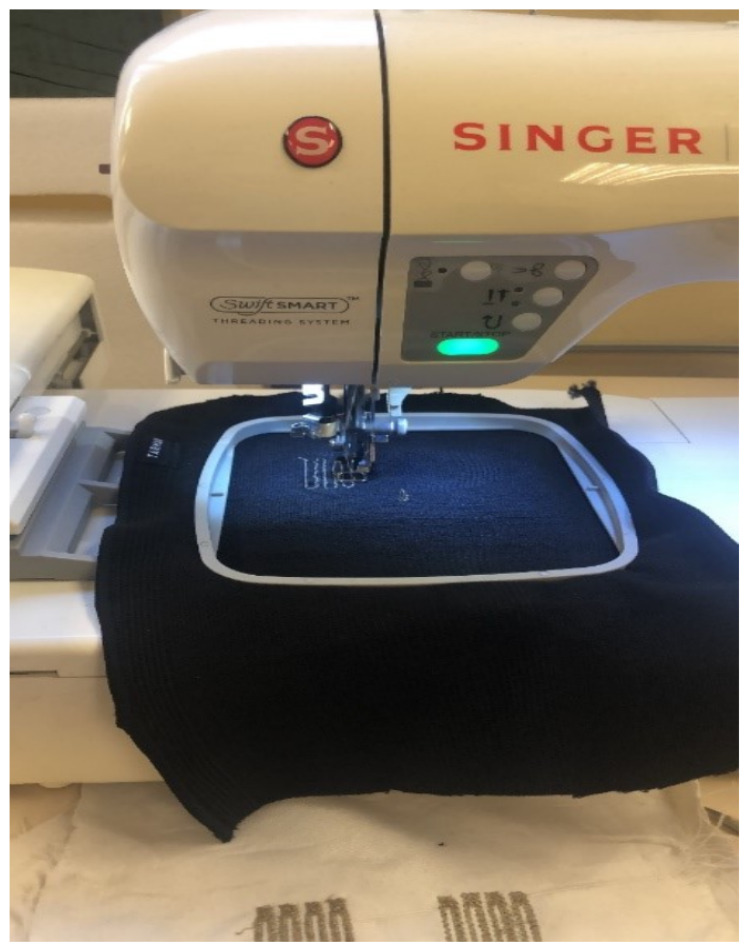
Embroidered-sensor manufacturing using a programmable embroidery machine [34].

Figure 3 shows the setup used for embroidered-sensor manufacturing, where conductive threads are precisely integrated into textile substrates to create flexible and repeatable sensing structures.

As summarised in Table 3, embroidery and stitching are particularly attractive for wearable, resistive sensors due to their compatibility with conventional textile manufacturing and their ability to precisely position sensing elements at ergonomically relevant locations.

In addition to the fabrication methods listed in Table 3, the structural design and material configuration of conductive yarns also play an important role in determining the performance of textile-based resistive sensors.

In textile-based resistive sensors, the structural configuration of conductive yarns can influence sensing performance. For example, higher braiding densities may reduce sensitivity while improving mechanical compliance and linearity. Additionally, the type of conductive yarn also influences the overall sensor behaviour. Compared with resistive sensors, capacitive textile sensors generally show lower sensitivity [77].

In textile-based systems, conductive or piezoresistive layers can also be deposited onto yarns through dip-coating processes [78], where threads are dipped into CNT- or graphene-based dispersions and subsequently dried or cured. This approach enables the formation of flexible, strain-sensitive conductive pathways directly integrated into fabrics.

### 6.2. Screen Printing

Screen printing is an advanced fabrication technique used in the production of flexible electronics. It involves applying conductive inks through a mesh screen onto a substrate, where pressure forces the ink through the mesh to form the desired pattern. This method is cost-effective and scalable, allowing printing of large-area sensor applications, including temperature and strain sensors, humidity sensors [12,79], as well as electrochemical devices [80].

**Figure 4:**
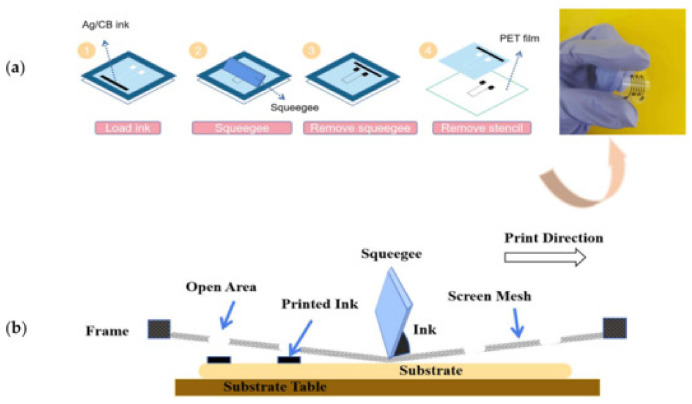
Screen-printing scheme: (**a**) schematic representation of the printing process; (**b**) illustration of the ink transfer and pattern formation on the substrate [32].

As shown in Figure 4, a schematic of the screen-printing process commonly applied in flexible electronics is presented [32]. The process involves a patterned mesh screen, a squeegee, conductive ink, and a substrate. During printing, the squeegee moves in a defined direction and applies mechanical pressure, forcing the ink through the open regions of the mesh onto the substrate surface. The closed areas of the screen block the ink, ensuring precise pattern transfer. After the screen is lifted, the deposited ink remains on the substrate, forming the designed structure.

### 6.3. Inkjet Printing

Inkjet printing is a precision-based technique used to deposit conductive inks or materials onto substrates to create flexible electronics. It includes two main systems: continuous inkjet (CIJ) and drop-on-demand (DOD), where CIJ continuously ejects droplets using an electric field, while DOD uses voltage pulses to control droplet release. This method enables high-resolution printing of conductive materials such as silver nanoparticles or conductive polymers (e.g., PEDOT:PSS) on flexible substrates, commonly used in developing flexible sensors [17].

**Figure 5:**
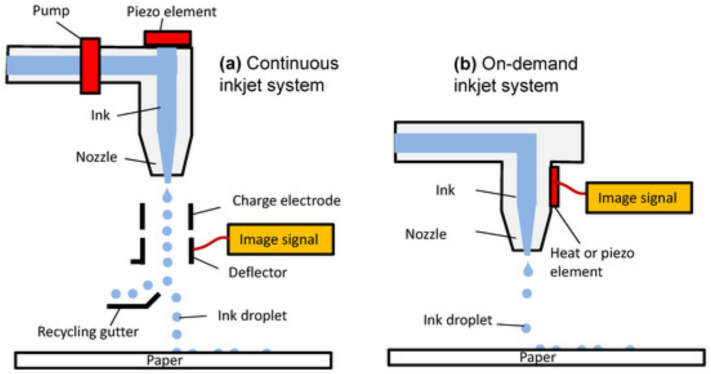
Inkjet-printing scheme [81].

Figure 5 shows a schematic representation of the inkjet printing process used in the fabrication of flexible electronic sensors [17,81]. In the continuous inkjet system, ink flows continuously through the nozzle, and droplets are electrically charged and deflected to form the desired pattern on the substrate. In the drop-on-demand system, droplets are generated only when an electrical signal activates a piezoelectric element or thermal actuator. The controlled ejection of microscale droplets enables precise material deposition and accurate pattern formation on the substrate.

Table 4 presents examples from selected studies demonstrating the use of printing technologies, particularly screen and inkjet printing, for fabricating resistive sensors on flexible substrates. The data highlights the suitability of these methods for wearable applications, where material flexibility, sensitivity, and low-cost fabrication are essential.

### 6.4. Direct Ink Writing

Direct ink writing (DIW) is an emerging additive manufacturing technique that offers a sustainable and efficient alternative to conventional subtractive methods for PCB fabrication. DIW involves the precise deposition of conductive inks, such as silver or copper nanoparticle-based formulations, directly onto substrates, guided by digital design data [16,58,84].

With its eco-friendly approach, rapid prototyping capabilities, and compatibility with complex electronic designs, DIW has gained significant attention. Studies demonstrate that PCBs manufactured through DIW achieve functional performance comparable to traditional methods while significantly reducing production time and costs [16]. The Voltera NOVA printer [84] enables rapid and low-cost production with precise control over sensor design, allowing for compact geometries and parallel patterns that ensure uniform strain distribution [84,85].

The studies listed in Table 5 illustrate how direct ink writing (DIW) and related additive manufacturing techniques are applied to fabricate flexible and functional sensing systems. One example highlights the use of DIW in embedded PCBs for aerospace applications, which enables precise and lightweight circuit designs [16]. Another demonstrates the printing of piezoresistive carbon inks for respiration monitoring, achieving a gauge factor of 22.5 [84]. Additionally, a fractal-patterned strain sensor produced via DIW shows high sensitivity and mechanical stretchability for soft robotics [86]. These examples confirm the versatility of DIW in developing stretchable and customised sensors for wearable, biomechanical, environmental, and aerospace applications.

In study [87], direct ink writing (DIW) with carbon nanotubes was used to create a flexible strain sensor with a gauge factor of around 100, showing good potential for wearable motion monitoring. Another study [88] achieved an even higher gauge factor (∼200) using a spalling-based method with conductive inks, which is promising for biomedical applications. These examples highlight how advanced fabrication techniques like DIW can improve the sensitivity of resistive sensors and emphasize the importance of further developing and studying resistive sensing technologies for ergonomic and wearable applications.

A summary and comparison of all the fabrication methods discussed in this section are presented in Table 6. The table outlines their resolution, suitability for wearable sensors, scalability, cost, and process complexity, providing a concise overview of the main advantages and limitations of each technique.

## 7. Ergonomics Applications

The advantages of flexible resistive sensors in ergonomics include their simple fabrication process, which is well-suited for wearable applications [89], and their ease of integration into textiles through simple printing processes paves the way for practical applications in smart healthcare systems, the Internet of Things (IoT), and human–machine interfaces. These sensors provide a versatile solution for monitoring respiration, arm motion, and other vital signals in both dry and wet environments [29]. Their importance in activity recognition [90], capturing typical human interaction forces [91], and sensor innovation emphasize the applicability of these sensors to human–robot interaction and ambient intelligence [91], as well as healthcare monitoring [66]. Key ergonomics aspects related to flexible resistive sensors include real-time posture and weight monitoring [24], and monitoring human body motions [39], such as joint bending [25]. Although many of the cited studies originate from wearable sensing or flexible electronics research, their sensing principles and integration strategies are directly relevant to ergonomic monitoring applications.

Table 7 presents representative studies applying flexible resistive strain and pressure sensors for posture analysis, motion monitoring, and ergonomic risk assessment in wearable systems. The reported studies indicate that resistive strain sensors are frequently used to monitor spinal or lumbar movements, enabling classification of sitting postures or trunk flexion [92,93,94]. In contrast, resistive pressure sensors are typically integrated into wearable interfaces such as gloves or insoles to evaluate external loads and force distribution during occupational activities [95,96].

### 7.1. Single Sensor Application

In ergonomic monitoring, single resistive sensors are often used to detect localised body movements or physiological signals. Fabric-based resistive strain sensors operate by incorporating conductive yarns or coated fibres into textile structures. As the fabric bends, stretches, or compresses in response to body movements, the electrical resistance of the conductive elements shifts. These resistance changes are then recorded and interpreted to identify motion or posture [21,43,67,68,71]. For real-world wearable use, such sensing configurations are commonly integrated directly into textile systems, providing comfortable, long-term ergonomic monitoring.

Table 8 presents representative examples of textile-based resistive sensors used for wearable sensing, highlighting their flexibility and suitability for ergonomic monitoring. A knitted sensor incorporating copper-based fibres reached a very high gauge factor (373–1560), suitable for capturing motion and physiological signals [67]. Another approach used conductive nanofibre yarns integrated into cotton fabric through hand-stitching, showing stable responses to elbow bending and breathing [43]. These examples underline the importance of fabrication techniques applicable to textiles in the development of soft, user-friendly sensors for ergonomic applications.

While single sensing elements provide accurate localised measurements of body motion or physiological signals, ergonomic evaluation in many scenarios requires spatial sensing capabilities, which motivates the use of resistive sensor arrays.

### 7.2. Array Sensor Application

In contrast to single sensing elements, resistive sensor arrays consist of multiple sensing units arranged in structured layouts, such as small clusters or larger grid patterns, enabling simultaneous measurement at several locations. Depending on the design, an array may include only a few sensors placed on key body points or many elements distributed across a surface.

This configuration allows for spatial detection of the strain or pressure distribution throughout the body or a surface, which is particularly useful for posture analysis, pressure mapping, and ergonomic risk evaluation.

Array sensors listed in Table 9 demonstrate strong adaptability for wearable and smart-environment applications. One sensor reached a gauge factor of approximately 68 by using GO-doped nanofibre yarns and electrospinning techniques, resulting in a stretchable, fabric-like structure suitable for physiological monitoring [97]. Another design enabled pressure mapping through embroidery and silicone rubber coating, enhancing sensitivity and allowing easy integration into textile substrates [47]. These examples highlight the strong potential of array-based resistive sensors for spatial pressure mapping and posture monitoring in ergonomic applications.

In addition to the textile and array-based configurations discussed above, piezoresistive sensing mechanisms are widely employed in both single-sensor and array-based wearable systems for motion and pressure monitoring.

Table 10 summarises representative piezoresistive sensors reported in wearable sensing studies, including both single sensing elements and array-based configurations relevant to ergonomic monitoring. The listed examples highlight different sensing configurations, targeted human-motion or physiological sensing applications, and key performance indicators such as sensitivity. Although these sensors employ diverse material compositions and fabrication strategies, the comparison emphasizes their suitability for human-centered sensing applications.

## 8. Discussion

Beyond the examples presented in this review, it is also important to note that, despite the advantages offered by flexible resistive sensors and the significant progress in their fabrication methods and performance, a critical gap remains between laboratory-based sensors and long-term practical applications. In wearable and ergonomic contexts, performance is affected not only by materials and fabrication methods, but also by failure modes such as resistance drift, calibration instability, substrate or ink adhesion, sensor attachment strategies for wearability, and long-term wearability constraints.

For example, resistance drift under cyclic loading can lead to signal instability and reduced calibration reliability. In addition, poor adhesion between conductive layers and flexible substrates may result in cracking or delamination, progressively degrading performance. While textile-based integration improves comfort, it can introduce variability in strain transfer and reduce measurement accuracy.

Based on these considerations, the following key limitations and trade-offs can be highlighted:

- Sensitivity and stability: High-sensitivity materials (e.g., CNTs, graphene) often show increased noise, hysteresis, and drift under cyclic loading.
- Flexibility and durability: Stretchable substrates (e.g., TPU, PDMS) improve comfort but may suffer from fatigue and long-term degradation.
- Textile integration and accuracy: Fabric-based sensors introduce non-uniform strain transfer, affecting precision and repeatability.
- Fabrication and control: Scalable methods (e.g., embroidery, DIW) reduce cost but limit control over microstructure and reproducibility.

Integrating ergonomic insights into the design of resistive flexible sensors is essential, not only for joint monitoring but also for broader posture and occupational health applications. Poor ergonomics in sedentary work, such as those experienced by professional drivers [26], and repetitive tasks in industrial sectors, such as aerospace assembly [24], create a high risk of musculoskeletal disorders (MSDs). Lower back pain already affects up to 23% of the global population [105], and approximately 53% of vehicle assembly workers report MSD symptoms, with the neck, shoulders, and lower back being the most affected areas [106]. In this context, flexible resistive sensors provide an opportunity to deliver real-time monitoring and feedback, offering a cost-effective and privacy-preserving alternative to traditional methods such as camera-based systems. This ergonomic integration emphasizes the potential of these sensors in addressing health and safety concerns in diverse environments.

Despite their promise, resistive flexible sensors face several technical and practical challenges. From a materials perspective, balancing mechanical flexibility, electrical sensitivity, stretchability, and long-term durability remains difficult [101]. Repeated strain often causes resistance drift, leading to signal instability and reduced accuracy. Ink formulation also plays a critical role, as viscosity, surface tension, and substrate compatibility directly influence conductivity and print quality. These problems are particularly relevant for direct ink writing (DIW) and related printing techniques, which require precise ink control to avoid uneven deposition or cracking. Furthermore, environmental conditions, including sweating, temperature changes, and complex body movements, reduce the stability of measurements in wearable settings. In wearable monitoring systems, wireless communication is often required for real-time data transmission. However, while improving user comfort, it also introduces challenges related to high power consumption, signal loss, and transmission delays. In addition, long-term use requires careful consideration of biocompatibility and user comfort, particularly for skin-contact applications [44]. Finally, real-time ergonomic applications demand accurate calibration, yet achieving stable and standardised calibration methods continues to be a major challenge.

## 9. Future Research Directions

Future research directions highlight several promising strategies to overcome the limitations discussed in this work. Material innovations, including nanocomposites, conductive polymers, and sustainable inks, can enhance conductivity and durability. Fabrication methods such as DIW and 3D printing allow for the creation of on-demand, personalised sensors, such as networks uniquely shaped for an athlete’s knee to prevent injury and improve performance [66]. System-level improvements include the use of electrode arrays and wireless data transmission to increase accuracy and usability in real-time applications [101]. Ergonomic integration requires combining these advances with accurate posture modelling [107], which strengthens calibration and supports preventive interventions in occupations involving repetitive movements and sustained postures. Additionally, artificial intelligence techniques, such as deep learning neural networks (DNNs), can improve posture recognition and contribute to vehicle design optimization [28]. Ensuring long-term usability will also require attention to ergonomic suitability, seamless textile integration, privacy-preserving wireless solutions, and user acceptance of wearable devices [27].

Another promising direction for future research is the integration of artificial intelligence (AI) with flexible resistive sensing systems. Recent studies have explored combining flexible strain sensors with machine learning algorithms to enable automatic classification of human motion patterns and posture recognition, highlighting the potential of intelligent wearable sensing systems [108,109,110,111]. Such AI-assisted systems can improve the interpretation of sensor signals, enabling automated posture assessment, activity recognition, and personalised ergonomic feedback.

Addressing these challenges demands interdisciplinary collaboration between ergonomics, electronics, materials science, and artificial intelligence. Such efforts will enable the development of adaptive, precise, and scalable ergonomic solutions applicable in healthcare, aerospace, and automotive sectors. By advancing both technical performance and ergonomic usability, resistive flexible sensors are positioned as a transformative technology for preventing musculoskeletal disorders and improving human comfort and safety. This perspective also provides a natural transition into the conclusion, which emphasizes the long-term potential of these sensors in ergonomic applications.

Flexible strain sensors have rapidly evolved in recent years, leveraging advanced printing technologies, especially direct ink writing (DIW), to deliver high-performance motion tracking devices for wearable applications. Material innovations, including nanocomposites with graphene, carbon nanotubes (CNTs), and metallic inks, have significantly improved sensitivity (with gauge factors from approximately 7 up to more than 400), while maintaining stretchability (commonly 10–50% or more) and durability across hundreds to thousands of cycles. Demonstrations on body regions such as hands, arms, legs, and the spine have shown the capability of these sensors to monitor motions ranging from subtle finger gestures to full knee bends.

In ergonomics, these devices are increasingly used for posture correction, repetitive motion analysis in workplaces, and real-time sports performance feedback, where they provide valuable insights into joint angles and strain. They also show promise in healthcare, particularly for rehabilitation, where printed sensors can track patients’ range of motion more comfortably than traditional bulky instruments. This review highlights the potential of flexible resistive sensors in ergonomic applications, particularly within wearable systems designed for healthcare, aerospace, and automotive industries.

By combining conductive materials such as carbon nanotubes, silver nanoparticles, and conductive polymers with fabrication techniques like DIW, inkjet printing, and embroidery, these sensors demonstrate exceptional adaptability and sensitivity. Their integration into textiles enables real-time monitoring of posture, joint movement, and physiological activity, addressing key ergonomic challenges such as musculoskeletal disorders and poor posture. However, challenges remain, including response variability, limited long-term durability, and the absence of standardised calibration protocols. Addressing these limitations should be a priority for future research.

In the future, the development of scalable, environmentally friendly fabrication methods, along with advances in wireless data transmission, will improve usability and reliability. Interdisciplinary collaboration between ergonomics, artificial intelligence, materials science, and electronics will be essential for building precise and adaptive systems. Moreover, long-term usability studies and standardised performance metrics are required to ensure consistent reliability in diverse applications. With these advances, flexible resistive sensors are well positioned to become a cornerstone technology in ergonomics, enabling injury prevention, health monitoring, and enhanced comfort in everyday life and industrial environments.

## Figures and Tables

**Table 1:** Examples of flexible substrates used in resistive sensor fabrication, along with key physical properties and typical applications.

Substrate	Stretchability	Typical Applications	Ref.
PDMS (Polydimethyl-siloxane)	40–120%	Soft, biocompatible substrate for skin-mounted sensors	[29,40,45]
TPU (Thermoplastic Polyurethane)	300–500%	Printed strain sensors for wearables	[37,38]
PU (Polyurethane)	50–200%	Substrate for CNT-based or inkjet-printed sensors	[35,47]
PET (Polyethylene Terephthalate)	<10%	Printable substrate, rigid support in hybrid sensors	[46]
PVA (Polyvinyl Alcohol)	Up to 50%	Hydrogel-based strain or humidity sensors	[29,42,48]
Polyamide/Elastodiene Blend (Shieldex textile)	Up to 65%	Embroidered sensors for joint monitoring (knee, elbow)	[34]
Cotton–Spandex Fabric	20–40%	Hand-stitched sensors for posture and motion sensing	[43]
Jeans Fabric	Low–Moderate (10–20%)	temperature sensors	[10]

**Table 2:** Selected examples of conductive materials used in resistive flexible sensors and their key properties.

Material	Conductivity (S/m)	Stretchability	Sensitivity	Applications	Ref.
Carbon Nanotubes (CNTs)	≈$10^{4}$–$10^{6}$	Up to ∼100%	Up to 200 [GF]	Flexible strain and pressure sensors	[35,38,42,57]
PEDOT:PSS	≈5–$10\times 10^{5}$	Up to ∼50%	≈1–10 [GF]	Printed biocompatible sensors	[56]
Silver Nanoparticles (AgNPs)	≈$10^{3}$–$10^{4}$	Conventional: <5%; engineered: up to ∼200–800%	≈10–50 [GF]	Inkjet or screen-printed, fibre-based, and advanced stretchable strain sensors	[15,21,49,50,51]
Graphene Nanoplatelets (GNPs)	$>10^{4}$	10–30%	∼50–100 [GF]	Wearable electronics, electronic skin	[46]
Carbon Black (CB)	≈$10^{-2}$–$10^{2}$	10–20%	∼10–30 [GF]	Cost-effective composites for motion detection	[39,49]
Polyaniline (PANI)	≈1–$10^{2}$	∼10–20%	Variable (1–50 [GF])	Humidity and strain sensors	[60]
Stainless Steel Threads	≈$10^{6}$	<5%	∼1–5 [GF]	Embroidered sensors in fabrics	[62]
Silver-coated Polyamide Yarn	≈$10^{3}$–$10^{4}$	<5%	∼24% ΔR	Embroidered sensors for joint monitoring	[34]
Copper Wire (0.4 mm)	≈$5.96\times 10^{7}$	Not stretchable	Temperature sensitive	embroidered temperature sensors	[10]
Silver Flakes (Ag flakes)	≈$10^{3}$–$10^{6}$	Up to ∼50–80%	∼1–10 [GF]	strain sensors for human motion monitoring	[54,55]

**Table 3:** Original research on textile-integrated resistive sensors for human motion monitoring based on embroidery or stitching techniques.

Sensor Type	Fabrication Method	Substrate	Target Body Part/Application	Ref.
Strain (resistive)	Stitching conductive fibres into wearable band	TPU-based fibre	Wrist bending and relaxation monitoring	[72]
Strain (resistive)	fibre sensor stitched into elastic garment	PDMS microfibre + textile	Knee-joint motion and squat monitoring	[73]
Strain (resistive)	Thread-based sensor sewn into textile	Conductive thread (textile)	Head and neck motion classification	[74]
Strain (resistive)	Conductive yarn embroidered into textile	Textile fabric	Upper-body posture and strain monitoring	[75]
Strain (resistive)	Spun conductive fibre integrated into textile	TPU fibre	Wearable object deformation sensing	[76]

**Table 4:** Selected examples of resistive flexible sensors fabricated using screen and inkjet printing methods.

Sensor Type	Use of Resistive Sensor	Sensitivity	Materials	Fabrication Method	Ref.
Stretchable Strain Sensor	Body motion tracking (e.g., arm flexion)	10% (flexion); 2% baseline drift	CNT ink	Screen Printing on PVA substrate	[42]
Textile Force Sensor (TFSR)	Finger movement and force sensing	4.9–7.1 MPa threshold pressure	Inkjet silver ink, TPU spacer	Inkjet printing + heat press	[17]
Screen-Printed Strain Sensor	Breathing monitoring (sports)	22 ± 2% (at 2.5 mm AP)	Carbon paste on TPU	Screen printing, encapsulation, heat press	[80]
Inkjet-Printed CNT Strain Sensor	Skin-mounted motion, vital sign monitoring	GF up to 400 (at 2.5% strain); 0.09 Pa^−1^ pressure sensitivity	SWCNT ink on PDMS with Ag electrodes	Inkjet printing (CNTs) + Screen printing (Ag)	[82]
Paper-based Resistive Tactile Sensor	Wearable pressure and motion sensing	6.67 kPa^−1^ (0.05–100 kPa); 1.19 kPa^−1^ (300–900 kPa)	CNT sensing layer, AgNP electrodes on mulberry paper	Inkjet printing	[83]

**Table 5:** Examples of DIW-based sensor systems, categorised by sensing mechanism, application domain, sensitivity, materials used, and fabrication methods.

Mechanism	Use of Resistive Sensor	Sensitivity	Materials	Fabrication Method	Ref.
Conductive PCB for Aerospace Applications	Embedded PCB systems	High precision, flexibility	Silver nanoparticle ink, copper conductive patterns	Direct Ink Writing (DIW)	[16]
Piezoresistive Strain Sensor	Respiration monitoring	GF [22.5]	Piezoresistive carbon ink, stretchable silver ink	Direct Write Extrusion	[84]
Temperature and Humidity Sensor	Environmental monitoring	High sensitivity, low hysteresis	Ag/AgCl ink, polyimide dielectric	Additive Printing (Voltera)	[85]
Fractal Flexible Strain Sensor	Motion detection, soft robotics	Highly sensitive	Graphene/PDMS ink	Direct Ink Writing (DIW)	[86]

**Table 6:** Comparison of main fabrication methods for resistive flexible sensors in terms of resolution, suitability for wearable applications, scalability, cost, and process complexity.

Fabrication Method	Resolution/ Accuracy	Suitability for Wearable Sensors	Scalability	Cost	Process Complexity
Textile-based (Embroidery, Knitting, Stitching)	Low–Medium (≈200–500 µm)	Excellent (breathable, comfortable, washable)	High (well-integrated in garment industry)	Low	Low–Medium (manual or automated)
Screen Printing	Medium (≈50–100 µm)	Good (requires encapsulation for durability)	High (scalable to large-area production)	Low	Low (mesh preparation, ink waste, limited resolution)
Inkjet Printing	High (≈20–50 µm)	Good (thin, flexible, patterned layers)	Medium (limited by ink formulation and nozzle clogging)	Medium	Medium (requires optimised inks and substrates)
Direct Ink Writing (DIW)	Medium–High (≈30–100 µm)	Excellent (customised designs, stretchable sensors)	Medium (good for rapid prototyping, less for mass production)	Medium	Medium–High (ink rheology and nozzle control critical)

**Table 7:** Representative original studies employing flexible resistive strain and pressure sensors for posture, motion, and ergonomic risk assessment.

Sensor Type	Fabrication Method	Substrate	Target Body Part	Ergonomic Application	Key Contribution	Ref.
Pressure (resistive)	Commercial FSR integration	Textile	Hand	Load detection during lifting	Real-time load estimation using tactile gloves	[95]
Strain (piezoresistive)	Nanocomposite fabrication	Elastomer	Spine	Sitting posture classification	Posture differentiation based on strain response	[92]
Strain (resistive)	Textile-integrated sensor	Textile	Lumbar	Trunk flexion assessment	Lumbar posture monitoring with fabric strain sensors	[93]
Strain (textile-based)	Embroidery (copper wire)	Elastic textile	Back	Back movement monitoring	Simple embroidered e-textile for posture sensing	[94]
Strain (hybrid resistive)	Conductive paint embedded in fabric	Textile (PPE)	Upper body	Awkward posture detection	Wearable extension sensor integrated into workwear	[4]
Pressure (resistive)	Commercial pressure array	Elastomer	Foot	Gait and balance risk analysis	Insole-based pressure sensing for ergonomic assessment	[96]

**Table 8:** Overview of fabric-based resistive strain sensors, including sensing configuration, applications, sensitivity, materials, and fabrication methods.

Sensing Configuration	Use of Resistive Sensor	Sensitivity	Materials	Fabrication Method	Ref.
Contact separation	Physiological signals motion activities	373–1560 GF	Acrylic/Copper Complex fibres (ACCFs), Spandex/Nylon Yarn, LYCRA fibre	Industrial knitting	[67]
Piezoresistance	Motion monitoring (walking, running, bending, sitting, standing)	2.5 GF	PBT, Carbon Ink, PDMS	Dip coating, layering	[21]
RTD	Wearable health monitoring	0.02 kΩ/°C	Jeans Fabric, Copper Wire, Cotton Thread	Embroidery	[10]
Resistive strain	Elbow bending, breathing, heartbeat	4 (from ΔR/R curve)	Conductive Composite Nanofibre Yarn (CCNY), Fabric 95% Cotton + 5% Spandex	Electro-spinning CCNY, hand-stitching	[43]
Piezoresistive	Respiratory monitoring	8%–109% (ΔR/R)	Conductive Yarn (Silver-plated and Unplated Nylon Twisted Around LYCRA), Fabric	Stitching	[68]

**Table 9:** Examples of array-based resistive sensors used for spatial pressure or strain monitoring.

Sensing Configuration	Use of Resistive Sensor	Sensitivity	Materials	Fabrication Method	Ref.
Conductive-network-based	Wearables, textiles, furniture interfaces, gesture recognition	500 Ω (light) to 70 Ω (strong pressure)	Copper core with carbon-based polymer coating	Wire drawing, annealing, quenching, dip coating	[98]
Single-layer fabric-based	Pressure mapping with detection of pressure magnitude and location	$\Delta R/R_{0}$: 0.5 (uncoated), 0.9 (coated)	Stainless steel fibres (0.012 mm), nylon substrate, silicone rubber coating	Embroidery stitching, rubber coating at contacts	[47]
Fabric-like stretchable	Respiration, facial motion, pulse, wearables	∼4.08 N^−1^; GF∼68	GO-doped PAN/PPY on elastic yarns	Electrospinning and in situ polymerization of PPY on GO-doped PAN nanofibre yarns	[97]
Resistive Pressure Sensor	Vehicle passenger monitoring (weight, position)	∼20 Ω under 45 kg	Linqstat (Velostat)	Lamination of copper electrodes and Velostat	[99]
Network contact resistance change	Smart mattress systems	∼0.1 kPa^−1^	Conductive polymers	Screen printing	[100]

**Table 10:** Representative examples of Piezoresistive sensors categorised according to sensing configuration, applications, sensitivity, materials, and fabrication methods.

Sensing Configuration	Use of Resistive Sensor	Sensitivity	Materials	Fabrication Method	Ref.
Resistance change due to strain	Monitoring knee-joint angle	Not provided	Polyurethane yarn, silver nanowires, graphene sheets	Knitting into textile (kneepad)	[66]
Force Sensing application	Different points on a surface for applied force	18.092 kPa^−1^	MW-CNT and PDMS	Sensor patch fabrication by casting	[101]
Tensile strain (strain resistive sensor)	Human motion, temp./strain monitoring	1.37 [GF]	Hydrogel	Ultrasound-assisted synthesis, layering technique	[102]
Resistive pressure	Pulse and joint movement detection	0.3 kPa^−1^–0.7 kPa^−1^	carbon nanotubes (VACNT) and (PDMS)	VACNTs grown on silicon, embedded and replicated	[45]
Applied mechanical pressure	Breath monitoring, muscle activity	0–14 kPa^−1^	Multi-filament conductive threads, aluminum sheet, laminated paper	Stitching conductive threads on fabric	[103]
Piezoresistive sensor arrays	Health monitoring: breath, motion, pressure	15.1 kPa^−1^	PDMS/CNTs	Micromolding	[63]
Piezoresistive pressure sensor	Human–machine interface applications	19.8 kPa^−1^	ACNT/G + Microstructured PDMS	CVD and leaf-based molding	[104]
Strain-induced resistance change	Wearable motion sensing	GF ∼100	CNTs	Direct ink writing	[87]
Spalling-induced flexible substrate	Biomedical monitoring	GF∼200	Conductive inks	Spalling fabrication technique	[88]
Pressure-induced conductive pathways	Robotics and prosthetics	GF∼120	Pressure-sensitive layers	Layer-by-layer fabrication	[48]
Deformation-induced resistance	Joint movement monitoring	GF∼25	Carbon black-filled silicone rubber	Mixing, molding, curing	[53]

## Data Availability

Not applicable.

## Supplementary Materials

The following supporting information can be downloaded at https://www.mdpi.com/article/10.3390/s26082563/s1, Table S1: PRISMA 2020 Checklist; Table S2: PRISMA 2020 Checklist, Cont’d.; Table S3: PRISMA 2020 Abstract Checklist.
